# A Concise History of the Principal Discoveries in Anatomy

**Published:** 1800-10

**Authors:** 


### *A Concise Hiſtory of the Principal Diſcoveries in Anatomy.*

[ Concluded from No. XIV. pp. 356—362. ]

§. 41. The *gloſſopharyngeus*, or our *ninth* pair, was generally conſidered in the ſixteenth century, as a branch of what was at that time called *the ſixth pair*, or our *eighth*, or *nervus vocalis*. FALLOPIUS was the firſt who diſtinguiſhed this nerve from the proper *nerve* of the voice, by evidently ſhowing its diſtribution to the tongue and fauces.* EUSTACHIUS aſſigned to his

* Obſervat. p. 406.

his ſixth pair three principal branches, viz. the *gloſſopharyngeus*, the *vocalis*, and the *acceſſorius Willisii*,* of which the *gloſſopharyngeus* was firſt figured by him.† Although VESALIUS knew the *nervus vocalis*, and its combination with the *lingualis medius*, or his ſeventh pair, and likewiſe *the recurrent branch*, he did not accurately enough purſue its courſe, fancying that it provided the bladder and uterus with nerves.‡ FALLOPIUS, however, evidently demonſtrated that beyond the liver and meſentery no inteſtine received any branch from the *nervus vocalis*.§ EUSTACHIUS likewiſe rightly figures the diſtribution of this nerve, and its final tranſition into the *intercostal nerve*,‖ but COLUMBUS¶ and GUIDI,** ſtill maintained the erroneous opinion of VESALIUS. The *nervus accessorius Willisii* was better known to the anatomiſts of the ſixteenth century than the *glossopharyngeus*, though it was taken for a branch of the *nervus vocalis*. VESALIUS deſcribes it as a branch of the ſixth pair, diſtributing itſelf to the muſcles of the fore and back neck;†† and likewiſe FALLOPIUS‡‡ and GUIDI.§§ EUSTACHIUS, however, has repreſented in his tables, the origin of this nerve from the third cervical nerve, its combination with the *eighth pair*, or *nervus vocalis*, and its progreſs to the *cleidomastoideus* and *cucullaris*, together with its communication with the third and fourth cervical nerve;‖‖ and KOYTER had even traced the roots of the acceſſory nerve to the fifth cervical nerve.¶¶

Our *twelfth pair*, (*the nerve of the tongue*, *lingualis medius*, *hypoglossus*,) was the ſeventh pair of the ancient anatomiſts. VESALIUS deſcribes indeed its origin from the corpora pyramidalia, its combination with the *nervus vocalis*, and diſtribution in the tongue; but in this deſcription he is very imperfect, committing at the ſame time the error of diſtributing this nerve to the *stylohyoideus*,*** which is not the caſe, as it only paſſes between the *hypoglossus*, to which it ſometimes imparts a ſmall branch, and the *stylohyoideus*.††† ETIENNE is acquainted with its communication with the firſt and ſecond cervical nerve,‡‡‡ and FALLOPIUS with its anaſtomoſes in the

---

* Oſſ. Exam. p. 205.

† Tab. xviii. fig. 2, (*mm*)

‡ Lib. vii. c. 9, p. 369.

§ Obs. p. 407

‖ Tab. xviii. fig. 2, (Mν)

¶ L. viii. p. 364.

** Lib. iii. p. 82.

†† Lib. vii. c. 9, p. 369.

‡‡ Obſ. p. 407.

§§ Lib. iii. p. 82.

‖‖ *Euſtache*, Tab. xviii. f. 1, 2, (ζηδι); fig. 2, (*efghik*); Tab. xix. fig. 2, (*fg.*); Tab. xx. fig. 2, (*abc*).

¶¶ *Koyter* Obſervat. p. 108.

*** *Veſal.* Lib. vii. c. 10, p. 372.

††† *Boehmer* de nono pare nervor. cerebr. § 43.

‡‡‡ *Stephan.* p. 249.

tongue, with the guſtatory nerve of the third branch of the fifth pair.* Du Laurens refutes the opinion of thoſe who adopt a combination between this and the *acouſtic nerve*, for explaining the co-exiſtency of deafneſs and dumbneſs.† Eustachius has given the firſt good repreſentation of its origin and progreſs.‡

§ 42. As for the *ſpinal nerves*, *thirty pair* of them were generally counted, viz. ſeven or eight *cervical nerves*, twelve nerves of the back, five of the loins, and ſix of the os ſacrum, or the falſe vertebræ. The old authors differ very much in the number of the cervical nerves, as ſome do not know the firſt cervical nerve, and, conſequently, have but ſeven pair; and others, who know it, think the ſeventh pair the laſt, calling our eighth, which comes out between the ſeventh vertebræ of the neck and the firſt of the back, the firſt back nerve of the bone. Berengar, however, differs from them by allowing eight cervical nerves, like the preſent anatomiſts.§ Zerbi had already found the true origin of the firſt cervical nerve,‖ and Vesalius deſcribes afterwards, with much accuracy, the bony canal or furrow in the *atlas*, through which it paſſes, diſtributing itſelf into the muſcles of the neck;¶ but he ſuppoſed that there were only *ſeven* cervical nerves, likewiſe taking the nerve, which iſſues between the ſeventh vertebra of the neck and the firſt of the back, for the firſt pair of the nerves of the back.** Etienne is entirely ignorant of our firſt cervical nerve, conſidering our ſecond pair as the firſt.†† Ingrassias deſcribes, much better than Vesalius, the origin and progreſs of the cervical nerves, particularly their ganglia and diſtribution into the fore and back branches. He thinks that the ſeventh pair combines itſelf ſometimes with the fourth, fifth, and ſixth cervical nerve.‡‡ Columbus too delineates with correctneſs the origin and progreſs of the firſt cervical nerve, and reproves Vesalius for having adopted twelve pair of the back, there being only eleven. It is probable that Columbus conſiders the laſt nerve of the back as the firſt of the loins.§§ Eustachius has a particular claim to be praiſed, for having firſt explained, by figures, the origin of the cervical nerves, and their communication with the *intercoſtal nerve.*‖‖

Koyter

* *Fallop.* Obſ. p. 407.

† *Laurent.* Hiſt. Anat. lib. xi. c. 11. p. 965.

‡ Tab. xviii. fig. 2. (10, 11, 12.)

§ Comment. in Mundin. f. 488. a.

‖ Anatom. p. 127.

¶ Lib. iv. c. 12. p. 379.

** Ibid. c. 13. p. 381.

†† *Stephan.* p. 73.

‡‡ Comment. in *Galen* de Oſſibus, p. 169, 171.

§§ Lib. viii. c. 4. p. 371. c. vi. p. 380.

‖‖ Tab. xviii. fig. 1, 3, 4, 5, (λ). Tab. xvii. fig. 2. (m).

KOYTER and others that deſcribe theſe nerves, have moſtly only quoted EUSTACHIUS.*

I have above mentioned, that the *intercoſtal nerve* was conſidered by many phyſicians of this century, as a continuation of the *nervus vagus*, or *vocalis*. ACHILLINI particularly,† VESALIUS ‡ and FALLOPIUS § conſidered the *intercoſtal nerve* as a branch of what was then the ſixth pair, and the Tables of EUSTACHIUS evidently ſhow his having been of the ſame opinion.‖ ZERBI, however,¶ and after him BERENGAR ** and MASSA,†† maintained, that this nerve was a continuation of the preſent fifth pair, becauſe they knew its combination with the *nervus vidianus*. ETIENNE was almoſt the only anatomiſt who took the *intercoſtal nerve* for a peculiar and ſeparate trunk.‡‡

§ 43. So far they were advanced at the beginning of the ſeventeenth century in the knowledge of the human body. It muſt be allowed, that a great deal is owing to the induſtry of the anatomiſts of thoſe times, and, particularly, to the immortal FALLOPIUS. However, the greateſt diſcovery that could take place in anatomy, the circulation of the blood, was not yet made. Of this, its cauſes and conſequences, we intend to lay before our readers at another time.

